# Dressed chicken as potential vehicle for spread of methicillin-resistant *Staphylococcus aureus* in Sokoto, Nigeria

**DOI:** 10.2144/fsoa-2020-0066

**Published:** 2020-08-24

**Authors:** Ibrahim A Musawa, Yusuf Yakubu, Bashiru Garba, Fatimah M Ballah, Hassan Abdurrahman Jibril, Abdulmalik S Bello, Mohammed Gaddafi Sani, Abubakar Farida

**Affiliations:** 1 Department of Veterinary Public Health & Preventive Medicine, Usmanu Danfodiyo University, Sokoto, Nigeria; 2 Department of Veterinary & Animal Sciences, Faculty of Health & Medical Sciences, University of Copenhagen, Denmark; 3 Department of Veterinary Microbiology, Usmanu Danfodiyo University, Sokoto, Nigeria; 4 Kebbi State Ministry of Animal Health, Husbandry & Fisheries; 5 Sokoto State Ministry of Animal Health & Fisheries Development

**Keywords:** carcass rinse, dressed chicken, Methicillin-resistant *Staphyococcus aureus*, Sokoto

## Abstract

**Aim:**

To evaluate the role of dressed chicken in the spread of methicillin-resistant *Staphylococcus aureus* (MRSA) in Sokoto, Nigeria.

**Materials & methods:**

190 chicken carcass rinsates were subjected to culture and biochemical analyses to isolate and identify MRSA. PCR was used to amplify mecA gene that is responsible for methicillin resistance.

**Results & conclusion:**

Culture and molecular analysis showed 19.5% (37/190) of the rinse had MRSA on oxacillin-resistance screening agar base (ORSAB) with 7.9% (15/190) possessing the mecA gene. Significant association (p = 0.044) exist between local-chicken and presence of MRSA, being twice more likely to have MRSA compared to exotic-chickens (odds ratio [OR] = 2.132). Results indicate possible role of dressed-chicken in the spread of MRSA. Authorities should regulate the sale and use of antibiotics by farmers, and enhance hygienic practices at slaughterhouses.

## Lay abstract

Methicillin-resistant *Staphylococcus aureus* (MRSA) is a multidrug-resistant pathogen that can be spread from animals to humans and *vice versa*. The occurrence of this pathogen in chicken is linked to indiscriminate antimicrobial usage by poultry farmers in a bid to improve productivity and maximize profit. This work was conducted to explore the potential role of dressed chicken in spreading MRSA among humans. It was found that due to the unhygienic nature with which chicken are dressed in the study area, such dressed chickens are contaminated with MRSA, thus can serve as vehicle for spread of MRSA among processors and consumers of chicken.

The pathogenic Methicillin-resistant *Staphylococcus aureus* (MRSA) is increasing in importance in hospitals, the community and livestock [1]. An estimated 5400 additional deaths have been attributed to it in the last few years, in addition to more than 1 million days of hospitalization [2]. Its development into MRSA occurred through the acquisition of *mecA* or *mecC*, which encodes the low-affinity PBP2a. As opposed to other PBPs, PBP2a remains active, with cell wall biosynthesis occurring at otherwise lethal β-lactam concentrations [1,3]. With various sources reported for MRSA strains, to distinguish its epidemiological groups, it is splitted into hospital-associated (HA-MRSA), community-associated (CA-MRSA) and livestock-associated MRSA (LA-MRSA) [4]. HA-MRSA was initially recognized in 1961. CA-MRSA was recognized as such in 1990. LA-MRSA has always been associated with animals and is linked to a jump from animals to humans [4,5]. *S. aureus* has been detected in several animal species and animal products [6,7], including poultry. One risk factor for colonization is intensity of contact with live animals, although colonization alone is not harmful [8]. LA-MRSA has been reported to cause severe infections in humans, including endocarditis [9].

Chicken is among the most affordable and consumed protein sources across the world, spanning both developed and developing nations. The Nigerian poultry industry comprises approximately 180 million birds making the country the second largest chicken producer in Africa after South Africa [10]. Nigeria produces about 454 billion tonnes of poultry meat with a per capita consumption at 2.5 kg per year [11]. Due to the absence of stringent hygienic measures imposed during the processing of poultry in slaughterhouses, poultry meat is prone to contamination from contaminated slaughterhouse environment, equipment and water used for dressing chickens. Pathogenic bacteria including *Staphylococcus aureus*, *E. coli* and *Salmonella* form part of the normal flora in the chicken gastrointestinal (GI) tract that could lead to food-borne diseases when accidentally consumed by humans [12].

*Staphylococcus aureus* is a gram-positive, coagulase-positive, facultative anaerobe and nonsporulating bacteria that mostly occur as an opportunistic pathogen. The bacteria are associated with asymptomatic skin and mucous membrane infections in humans, animals, and also as an environmental contaminant [13] with some strains causing disease through the production of various toxins and enzymes [14].

Poultry and poultry products are important vehicles for the transmission of food-borne pathogens [15]. *S. aureus* has been found to colonize the mucosal membranes in domestic and commercially raised poultry birds. The first report on the occurrence of MRSA in poultry was from South Korea [16]. Thereafter, several researchers have isolated MRSA from live birds as well as from poultry meat [17]. Traditionally, chickens are usually reared in close proximity to human habitation, especially in resource-poor countries, including Nigeria. Hence, they are believed to play important roles leading to contamination of the environment, as well as serving as vehicles for the spread of these pathogens to humans during handling of live birds or consumption of contaminated meat and other poultry products. Isolation of MRSA in the nostrils of meat sellers screened in a study conducted by Kwoji *et al.* [18] indicates potential dangers to the sellers, their families and the general public.

Several studies have been conducted worldwide that established carriage of MRSA in poultry and poultry products; there is however little information in the study area. Moreover, there is need to assess the potential spread of MRSA through dressed chicken slaughtered and sold in the study area.

## Materials & methods

### Study area

In this study, sampling was conducted at the chicken slaughter slab located at the live bird market in Sokoto township, Nigeria. Sokoto is the capital of Sokoto State, located in the extreme northwestern Nigeria. It shares boundaries with the Niger Republic to the north, Kebbi State to the west and southwest, and Zamfara State to the East.

### Sample size determination

$$n=\frac{Z^{2}pq^{2}}{d^{2}}$$

where d = acceptable margin of error = 5%, Z = level of confidence = 1.96 SE at 95% CI, p = prevalence from previous study = 14.29% [18], q = complimentary probability of p = 1-p from above formula; d = 0.0025, Z^2^ = 3.8416, p = 0.1429, q = 0.8571.

$$\text{n}=\frac{\text{3}\text{.8416}\times \text{0}\text{.1429}\times \text{0}\text{.8571}}{\text{0}\text{.0025}}$$

n = 188.21

Therefore, a minimum of 188 samples are required to achieve a representation of the population. However, the sample size was rounded up to 190 to increase rate of detection.

### Sample collection

Methods described and adapted by De Cesare *et al.* [19] were used to collect poultry rinsate. Whole-dressed carcass was immersed in sterile polythene bag containing predetermined volume of distilled water. Carcasses with weights ranging from 2.1 to 2.7 kg were rinsed with 300 ml of sterile water, while carcasses ranging from 1.2 to 1.9 kg were rinsed with 200 ml of sterile water.

The polythene bag was sealed and then shaken vigorously for about 1 min before the bag was loosened, and the chicken is removed. About 15–20 ml of the rinsate was aliquoted into a sterile-capped sample bottle; properly labeled and were transported on ice packs to the Veterinary Bacterial Zoonoses Laboratory of Usmanu Danfodiyo University, Sokoto, for immediate analysis.

### Culture & isolation

#### Culture on mannitol salt agar

An inoculum was taken using a sterile wire loop and streaked on mannitol salt agar (MSA; Oxoid^®^, Basingstoke, UK) and incubated at 37°C for 24 h. Isolates that produced colonies exhibiting characteristic deep golden yellow color suggestive of *Staphylococcus aureus* [20] were identified.

#### Biochemical characterization

Coagulase and Catalase tests for identification of *S. aureus* were performed as described by Cheesebrough [21].

#### Subculture on oxacillin-resistance screening agar base

Presumptive colonies of *Staphylococcus aureus* were picked and subcultured onto oxacillin-resistance screening agar base (ORSAB, Oxoid^®^ UK) and incubated for 24 h at 37°C. Positive isolates were identified by their deep blue color appearance due to Mannitol fermentation [21]. Thereafter, positive isolates were transferred onto nutrient agar in preparation for genomic DNA extraction.

#### DNA extraction

Genomic DNA was extracted from the fresh cultures by the boiling method, as described by Queipo-Ortuño *et al.* [22] and Dilhari *et al.* [23]. Briefly, two loopful of bacterial colonies freshly grown on nutrient agar were suspended in 100 μl of sterile distilled water in a 1.5-ml microcentrifuge tube. The suspension was first incubated for 5 min at room temperature before boiling at 96°C for 10 min in a water bath. The boiled suspension was then centrifuged at 13,000 rpm for 5 min, and the supernatant was decanted in a new microcentrifuge tube and used as the DNA template.

#### Amplification of *mecA* gene

The extracted DNA was subjected to PCR to amplify the 163-bp fragment of the *mecA* gene using the following primer sequences as reported by Mehrotra *et al.* [24].

$$mecA\;\text{Forward-}\;{\text{5}}^{\prime }\;\text{ACTGCTATCCACCCTCAAAC}\;{\text{3}}^{\prime }$$

$$mecA\;\text{Reverse}\;{\text{5}}^{\prime }\;\text{CTGGTGAAGTTGTAATCTG}\;{\text{3}}^{\prime }$$

The PCR amplification was done in a 25-μl reaction volume. Four microliters of genomic DNA was added to each reaction tube containing 12.5 μl of PCR Master Mix (Qiagen^®^, Hilden, Germany), 0.5 μl each of the forward and reverse primers, 2 μl of Coral load (Gel-loading dye, Qiagen) and the final volume was adjusted to 25 μl by adding 1.5 μl of RNase-free water (Qiagen). The reaction protocol for the PCR amplification entails an initial denaturation temperature of 94°C for 5 min, followed by 35 cycles of denaturation at 94°C for 1 min, annealing at 57°C for 2 min, extension at 72°C for 1 min and a final extension at 72°C for 5 min. The PCR amplification was accomplished with the aid of a GeneAmp PCR System 9700 Thermal Cycler.

#### Agarose gel electrophoresis

In order to view the amplified PCR fragment, a 1.5% agarose gel was prepared by dissolving 1.5 g of agarose powder (L.E. Agarose CSL-AG500, Cleaver Scientific Ltd, Warwickshire, UK) in 100 ml of 1× Tris-Borate-EDTA buffer, using a microwave oven. About 5 μl of ethidium bromide (nucleic acid-staining solution) was added into the molten agarose before pouring into a gel caster, a ten-well comb was inserted and then allowed to solidify at room temperature. After solidification of the agarose, the comb was removed, creating wells and then placed inside an electrophoresis tank (BioRad^®^, UK), and the tank was flooded with 0.5× Tris-Borate-EDTA buffer to the recommended level. Finally, 8 μl of the amplicons was loaded in the wells of the submerged gel and electrophoresed at 90 V for 40 min. The electrophoresed gel was viewed using a BioRad Gel Doc^®^ imager, and the images were captured, labeled and saved accordingly. Amplicons that have the band size of 163 bp were considered to contain the *mecA* gene.

### Data analysis

Data were initially stored in Microsoft Excel 2016 (Microsoft Corporation, USA) later exported to Statistical Package for Social Sciences (SPSS) version 22 (IBM, USA) for inferential statistics. The results obtained in this study were presented in tables, charts and percentages. Chi-square test was used to determine association between occurrence of MRSA and categorical variable (type of bird). Values of p < 0.05 were considered statistical significance.

## Results

A total of 190 samples of chicken rinsate were screened for the presence of *S. aureus*. Out of those, 24.2% (46/190) was positive on MSA, comprising 20 rinsate from commercially raised birds (exotic birds; broilers) and 26 rinsate from local chicken raised in backyards (extensive system) for subsistence. Presumptive positive colonies were subjected to Catalase and Coagulase test. From this, 22.6% (43/190) was catalase positive, while 22.1% (42/190) were coagulase positive.

All the 42 isolates that were positive on both catalase and coagulase tests and two other local chicken isolates that are coagulase negative were then subcultured on ORSAB, of which 19.5% (37/190) was positive (13 from broiler rinse and 24 from local chicken rinse; Table 1).

**Table 1.** Biochemical and cultural identification of isolates obtained from poultry carcass rinsate in Sokoto, Nigeria.

Type of chicken	No. of samples	No. positive on MSA	No. positive by biochemical analysis	No. (%) positive on ORSAB	p-value	X^2^	OR	95% CI
Exotic (Broiler)	95	20	20	13 (13.7%)	0.044	4.061	2.132	1.011–4.496
Local	95	26	22	24 (25.3%)				
Total	190	46	42	37 (19.5%)				

MSA: Mannitol salt agar; OR: Odds ratio; ORSAB: Oxacillin-resistance screening agar base.

**Figure 1.**
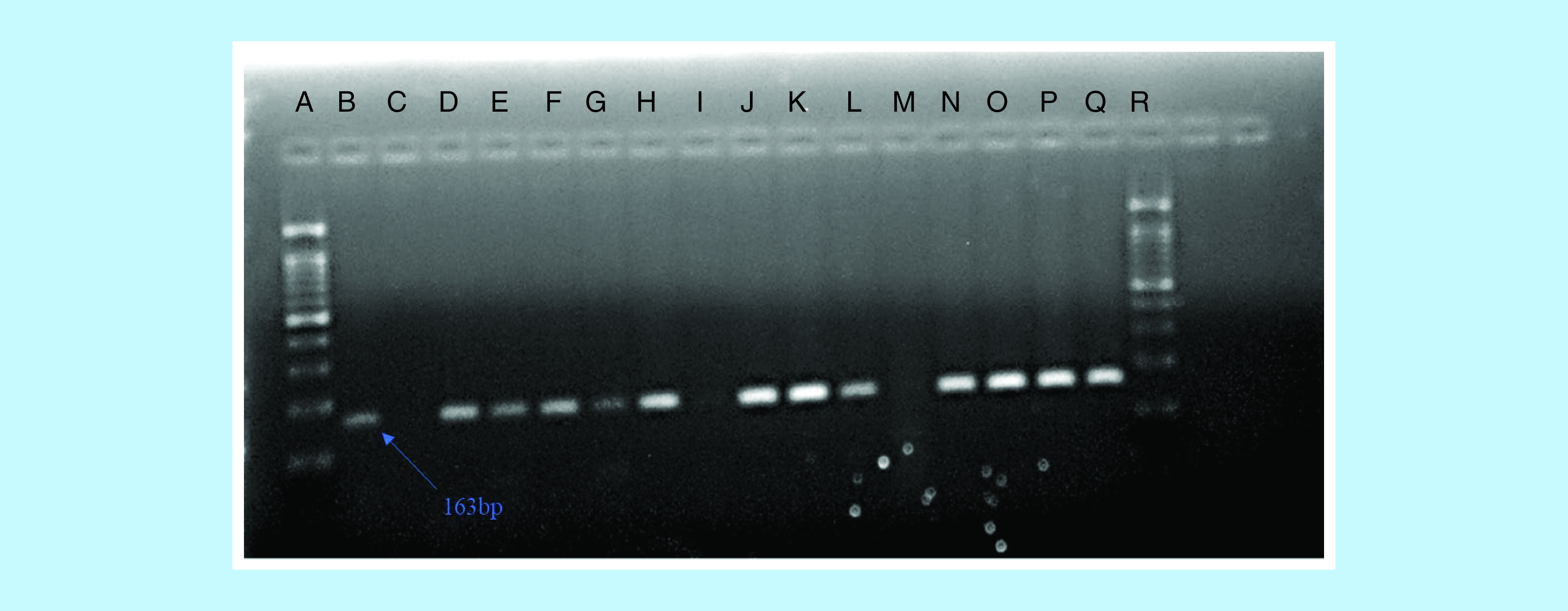
Agarose gel electrophoresis image. Showing 100-bp molecular weight ladder **(A & R)**, positive control **(B)**, negative control **(C)** and samples positive for *mecA* gene **(D–H, J–L & N–Q)**.

Similarly, of the 37 samples positive on ORSAB that were subjected to PCR amplification, the 163-bp *mecA* gene, as shown on Figure 1, was detected in 40.5% (15/37) of the isolates giving rise to an overall detection rate of 7.9% (15/190). Of the 15 *mecA*-positive isolates, 40% (6/15) was from broiler rinse, while 60% (9/15) was from the local chicken rinse (Table 2).

**Table 2.** Identification of methicillin-resistant *Staphylococcus aureus* by PCR amplification of the *mecA* gene.

Type of chicken	No. of samples	No. (%) positive	p-value	X^2^	OR	95% CI
Exotic (Broiler)	95	6 (6.3%)	0.420	0.651	1.552	0.530–4.547
Local	95	9 (9.5%)				
Total	190	15 (7.9%)				

OR: Odds ratio.

## Discussion

Unhygienic processing of poultry meat creates an environment that favors the growth and proliferation of numerous pathogenic microbes, including *S. aureus*. Screening to identify *S. aureus* has been in use to monitor hygienic conditions during slaughter [25,26]. The inherent ability of *S. aureus* to express various survival characteristics including resistance to chlorine [27] and the fact that meat provides an ideal medium for microbial growth [28] is an indication that meat can be a vehicle for the spread of *S. aureus* among humans.

In the present study, an overall prevalence of *S. aureus* recorded for chicken carcass rinse was 21.1% on selective media. However, 19.5% was recorded when the samples were screened for MRSA. The significance of this finding is that although the agent was isolated from chicken rinse, it still poses a zoonotic risk for the humans handling the poultry [16,29,30].

Of the 37 methicillin-resistant isolates cultured on ORSAB, only 15 were found to harbor the *mecA* gene, this may be because apart from *mecA* gene *mecC* gene can also confer antimicrobial resistance to *S. aureus* [31] and since our PCR is only targeting *mecA* gene, perhaps the other ORSAB-positive isolates are harboring the *mecC* gene.

The detection of the *mecA* gene in 7.9% of the samples further indicates the risk faced by poultry handlers and consumers. Risks such as the rapid spread of MRSA within the society and an exponential increase number of outbreaks due to MRSA cannot be ignored. The detected prevalence of 7.9% is very much close to 7.8 and 7.4% reported in Taiwan and China, respectively [1,26]. The closeness of these prevalences with our reported prevalence may not necessarily indicate the same burden because both Taiwan and China have better sanitary conditions in their meat-processing plants than is obtained in our study area. Also, both studies were conducted over longer periods of time (2 and 5 years for Taiwan and China, respectively) allowing for more MRSA to be detected, compared with 6 weeks within which our study was conducted.

Previous investigations conducted in Nigeria reported varying prevalence of MRSA in meat to be 10% in north-east [31] and 20% in Benin City [32], but a more recent investigation of MRSA from swabs collected from village chicken in Maiduguri revealed a prevalence of 32.60% [33]. While these reports have prevalence higher than our prevalence rate, the burden may actually be the same. This is because, in all those reports, ORSAB was used for final confirmation of MRSA while in our study, we went further to use the PCR technique that is more sensitive in discriminating MRSA, hence the lower prevalence recorded.

Our investigation found significant association (p = 0.044 [95% CI: 1.011–4.496]) to exist between the occurrence of MRSA and the type of chicken. Local chickens were found to be two-times more likely to be MRSA positive (OR: 2.132) than the exotic chicken. Although the exotic chickens that are intensively managed may be more exposed to treatment with antibiotics, the local chickens may also be subjected to subtherapeutic doses over an incomplete regimen leading to the development of drug-resistant microbes [33]. Also, local chickens are raised as scavengers and for longer periods before reaching market weight giving them more time to interact and gain access to MRSA in the environment [33].

## Conclusion

In the face of global food security challenges and the constant increase in poultry meat consumption worldwide, ensuring the microbial safety of poultry carcasses has become essential. The high-level detection of MRSA in poultry carcass wash in this study may signify the unwholesomeness of poultry meats produced from the Sokoto live bird market and the potential risk it presents to the consumers. The emergence of MRSA and community-acquired MRSA is largely attributed to the unregulated use of drugs for either treatment or as growth promoters in livestock. Therefore, further studies are recommended in order to understand the burden of the disease caused with a view to instituting control measures including stringent legislation on the use of drugs as well as hygienic meat-processing practices.

## Future perspective

The fact that 8 years back MRSA was causing more deaths than HIV/AIDS in the USA highlights how developing countries like Nigeria should take MRSA with utmost seriousness. The indiscriminate use of antimicrobials by poultry farmers and the unhygienic nature in poultry slaughterhouses needs to be addressed by governments, otherwise morbidity and mortality due to MRSA will continue to increase. Therefore, future research should be focused on how to mitigate the effect of antimicrobial resistance in animals through techniques that will ensure zero usage of antimicrobials in livestock farming. This can be achieved through development of chicken breeds that are resistant to infection by multidrug-resistant organisms or through development of newer drugs that the currently known antimicrobial resistance genes cannot confer resistance to.

**Summary points**

- Methicillin-resistant *Staphylococcus aureus* (MRSA) is found in both local and exotic chicken within the study area.
- MRSA in the gastrointestinal tract of chickens can contaminate the water used in processing chicken in slaughterhouses.
- MRSA is also seen to contaminate both local and exotic chicken during dressing in the study area.
- Contaminated dressed chicken can serve as a vehicle for transmission of MRSA to people processing or consuming these dressed chickens in the study area.
- Authorities need to enforce strict compliance with hygienic measures during slaughter and processing of chicken in the study area.
- Authorities need to regulate the indiscriminate use for antimicrobials in poultry farms within the study area so as to reduce the rate at which pathogens become resistant to these antimicrobials.
